# Percutaneous Closure of Left Atrial Appendage significantly affects Lipidome Metabolism

**DOI:** 10.1038/s41598-018-23935-w

**Published:** 2018-04-12

**Authors:** G. Yücel, M. Behnes, C. Barth, A. Wenke, B. Sartorius, K. Mashayekhi, B. Yazdani, T. Bertsch, J. Rusnak, A. Saleh, U. Hoffmann, C. Fastner, S. Lang, X. Zhou, K. Sattler, M. Borggrefe, I. Akin

**Affiliations:** 1 First Department of Medicine, Faculty of Medicine, University Medical Centre Mannheim (UMM), University of Heidelberg, Mannheim, Germany; 2 DZHK (German Center for Cardiovascular Research), Partner Site, Heidelberg-Mannheim, Mannheim, Germany; 3 Clinic for Cardiology and Angiology II, Universitäts-Herzzentrum Freiburg - Bad Krozingen, Bad Krozingen, Germany; 4 Fifth Department of Medicine, Faculty of Medicine, University Medical Centre Mannheim (UMM), University of Heidelberg, Mannheim, Germany; 5 Institute of Clinical Chemistry, Laboratory Medicine and Transfusion Medicine, General Hospital Nuremberg and Paracelsus Medical University, Nuremberg, Germany

## Abstract

Patients with non-valvular atrial fibrillation (AF) and a high risk for oral anticoagulation can be treated by percutaneous implantation of left atrial appendage occlusion devices (LAAC) to reduce the risk of cardio-embolic stroke. This study evaluates whether LAAC may influence lipid metabolism, which has never been investigated before. Patients with successful LAAC were included consecutively. Venous peripheral blood samples of patients were collected immediately before (T0, baseline) and 6 months after (T1, mid-term) LAAC. A targeted metabolomics approach based on electrospray ionization liquid chromatography–mass spectrometry (ESI-LC-MS/MS) and MS/MS measurements was performed. A total of 34 lipids revealed a significant change from baseline to mid-term follow-up after successful LAAC. Subgroup analysis revealed confounding influence by gender, age, diabetes mellitus type II, body mass index, left ventricular ejection fraction, creatinine and NT-proBNP. After multivariable adjustment within logistic regression models, these 34 lipids were still significantly altered after LAAC. Successful percutaneous LAAC may affect lipid metabolism and thereby may potentially affect pro-atherogenic and cardio-toxic effects.

## Introduction

Atrial fibrillation (AF) is a common supraventricular arrhythmia. While anticoagulation is effective in preventing stroke, the risk of major hemorrhage may be increased especially in older patients^1,2^. The left atrial appendage (LAA) is the main cardiac anatomic structure for thrombus formation. Stroke prevention in patients with AF and high risk for bleeding still remains a challenge^3^. The percutaneous closure of the LAA with occlusion devices (LAAC) is an established interventional treatment for reducing both stroke and bleeding risk in these patients^4–6^.

Besides its hemodynamic role for volume filling within the cardiac cycle, the LAA and atrial cardiomyocytes are presumed to reveal metabolic and endocrinological functions, of which the production of atrial natriuretic peptide (ANP) has been studied mostly^7,8^. Physiological alterations such as volume loading may effect the atrial production of ANP^9^, whereas the influence of the left atrium or LAA on systemic metabolism has rarely been investigated.

Metabolome is the common term for the global collection of metabolites excluding nucleic acids or proteins. Metabolomics (the more common term) define the biological response of a living system to a stimulus, involving the identification and measurement of metabolites in biological samples through several analytical methods such as chromatography or mass spectrometry. Lipid metabolism is also described as lipidome, which includes several defined lipid subclasses including phosphatidylcholines (PC), lyso-phosphatidylcholines (lysoPC) or sphingomyelins (SM)^10,11^.

Therefore, the present study investigates whether successful LAAC treatment in patients with non-valvular AF may affect lipidome pathways.

## Methods

The “Left Atrial Appendage Occlusion and Biomarker Evaluation” (LABEL) study (ClinicalTrials.gov Identifier: NCT02985463) is a single-centre, prospective, observational non-randomized study including patients being eligible for percutaneous LAA closure according to European guidelines^12^. All patients presented with non-valvular AF, a CHA2DS2-Vasc score ≥2, a HAS-Bled score ≥3 and a contraindication for the therapy with oral anticoagulants, i.e. major or recurring bleedings. Exclusion criteria included age <18 years, congestive heart failure classified as NYHA IV, catheter ablation of AF within 30 days prior to planned intervention, myocardial infarction within the last 3 months, atrial septum defect (ASD) or implanted ASD occluder, mechanical heart valves, status after heart transplant, symptomatic carotid artery stenosis, transient ischemic attack or stroke within 3 months, existing or planned pregnancy, acute infection or planned thrombus at the day of planned implantation. Patients with unsuccessful LAAC as being assessed by transesophageal echocardiography (TEE) at mid-term follow-up (i.e. 6 months), for instance by evidence of incomplete LAAC or significant per-device leaks were excluded. The study was carried out according to the declaration of Helsinki and was approved by the medical ethics committee II of the Faculty of Medicine Mannheim, University of Heidelberg, Germany. Written informed consent was obtained by all patients or their legal representative.

### LAAC and blood sampling

LAAC was performed using either the Watchman (Boston Scientific, Marlbrough, MA, USA) or Amplatzer Amulet (St. Jude Medical, St. Paul, MN, USA) device. Blood samples were taken by venous puncture within 24 hours prior to cardiac intervention (T0). Secondary blood samples were taken at least 6 months later (i.e. mid-term) (T1). Successful LAAC was confirmed by TEE during index procedure, as well as at mid-term follow-up by TEE and cardiac computed tomography angiography (CCTA).

Venous blood samples were taken from each patient and collected into serum monovettes® and EDTA monovettes® and centrifuged at 2500 × g for 10 minutes at 20 °C. The aliquoted samples were cooled down with liquid nitrogen before being stored at −80 °C until analysis. The whole processing took part within two hours after blood extraction.

### Metabolite Analysis

A targeted metabolomics approach based on electrospray ionization liquid chromatography–mass spectrometry (ESI-LC-MS/MS) and MS/MS measurements was performed using the AbsoluteIDQ™ p180 Kit (BIOCRATES Life Sciences AG, Innsbruck, Austria). The assay allows simultaneous quantification of in total 188 metabolites out of 10 µL plasma samples, including amino acids, biogenic amines, glycerophospholipids, sphingolipids and the sum of hexoses. Analyses were carried out on a QTRAP 4000 System (Sciex Deutschland GmbH, Darmstadt, Germany) and a Thermo TSQ (ThermoFisher Scientific Waltham USA). For the evaluation of metabolite concentrations, internal standards served as a reference. BIOCRATES MetIDQ™ software was used for the processing and technical validation of the metabolite data.

### Statistical analysis

To exclude metabolites of which concentration values are below LOD, a general cleaning of the data set based on an 80% rule was performed. Remaining values below LOD in the data set were then imputed applying a logspline imputation method and the resulting data set was log2 transformed^13,14^.

Principal Component Analysis (PCA), Partial Least Squares Discrimination Analysis (PLS-DA) and Hierarchical Cluster Analysis (HCA) were used as supervised and unsupervised multivariate approaches^15^. To compare significant differences, data were subjected to a student’s t-test or repeated measures ANOVA (rANOVA). To control the False-Discovery-Rate (FDR) during multiple comparisons an adjusted p-value (Benjamini-Hochberg correction) was additionally calculated^16^.

A regression analysis based on a linear mixed effect model was applied for the evaluation of significant metabolites dependent on all seven subgroups (gender, age, diabetes mellitus type II (DM), body mass index (BMI), left ventricular ejection fraction (LVEF), creatinine, aminoterminal pro-B type natriuretic peptide (NT-proBNP)). The median within the study population or internal standards were used to detect the cut-off points for subgroup analysis. Statistical analysis was performed using R-Studio^17^. A p value of <0.05 was considered significant for all statistical analyses.

## Results

### Study population

A total of 44 patients with successful interventional LAAC were included into the present study. Median CHA2DS2-VASc score and HAS-BLED score was 4 (interquartile range (IQR) 3–5 and 3–4.3, respectively). Baseline characteristics of the patients are outlined in Table 1. Patients’ medication influencing volume status, electrolytes, sympathetic activation, blood sugar or lipid status, such as diuretics, beta-blockers, ACE/aldosterone antagonists, insulin, biguanides or statins, did not change in type or dosage in between baseline and follow up. Clinical parameters like body weight were in steady state during follow up.

**Table 1:** Baseline characteristics of 44 patients with successful LAAC and biomarker evaluation.

**Demographics**	
Sex, male n (%)	30 (68.2)
Age, y (IQR)	77 (75.8–83)
Height, cm (IQR)	170.3 (165–176)
Weight, kg (IQR)	81.8 (69.8–92)
BMI, kg/m^2^ (IQR)	28.1 (24.7–32.7)
**Cardiovascular risk factors, n (%)**	
Hypertension	42 (95.4)
Diabetes mellitus	16 (36.7)
Hypercholesterinemia	22 (50)
**Medical history, n (%)**	
Atrial fibrillation, n (%)	
paroxysmal	24 (54.5)
persistent	6 (13.5)
permanent	14 (31.8)
LVEF, n (%)	
normal (>55%)	34 (77.2)
mild (45–54%)	4 (9.1)
moderate (30–44%)	4 (9.1)
severe (<30%)	2 (2.5)
NT-proBNP, ng/l (IQR)	1038.5 (499.7–146.0)
Prior PVI, n (%)	4 (9.1)
TIA, n (%)	3 (6.8)
Stroke, n (%)	7 (15.9)
Coronary artery disease, n (%)	25 (56.8)
Prior myocardial infarction, n (%)	10 (22.7)
Heart failure, n (%)	10 (22.7)
Peripheral vascular disease, n (%)	4 (9.1)
Chronic kidney disease, n (%)	18 (40.1)
Creatinine, mg/dl (IQR)	1.05 (0.94–1.25)
MDRD-GFR, ml/min (IQR)	65.5 (52.7–79.7)
Chronic liver disease, n (%)	3 (6.8)
Prior bleeding, n (%)	34 (77.3)
**CHA**_**2**_ **DS**_**2**_ **-VASc score (IQR)**	4 (3–5)
**HAS-BLED score (IQR)**	4 (3–4.3)
**Events at mid-term follow-up, n (%)**	
Acute myocardial infarction	1 (2.3)
Stroke	0
Pulmonary embolism	1 (2.3)
Bleeding according to BARC score	8 (18.2)
Type 1	1 (2.3)
Type 2	5 (11.4)
Type 3a	2 (4.5)
≥Type 3b	0
Rehospitalization	24 (54.5)
Cardiovascular	14 (31.8)
Bleeding	7 (15.9)
Orthopedic/traumatic	3 (6.8)
Dermatological	1 (2.2)
Renal	1 (2.2)

Values are given as median (25th and 75th percentiles) or total numbers (percentage). AF = atrial fibrillation, LVEF = left ventricular ejection fraction, PVI = pulmonary vein isolation, TIA = transient ischemic attack, MDRD-GFR = Modification of Diet in Renal Disease-glomerular filtration rate. AMI = acute myocardial infarction. BARC-Score: Standardized bleeding definitions for cardiovascular clinical trials according to the consensus report from the Bleeding Academic Research Consortium^39^.

### Hierarchical cluster and lipid-subclass alterations

**Figure 1:**
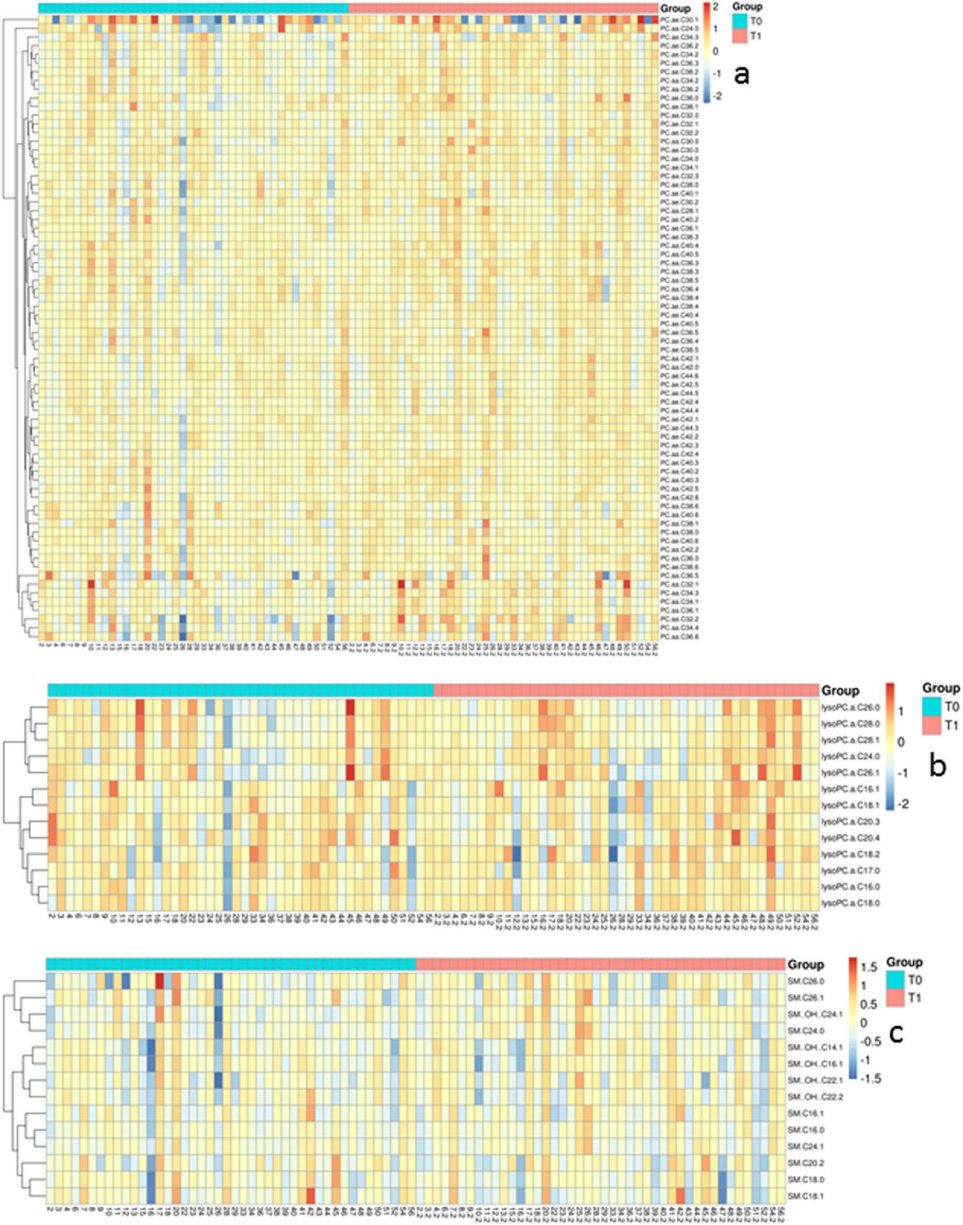
Hierarchical cluster analysis illustrating the results of lipid metabolome changes in patients undergoing LAAC therapy. The upper x-axis is showing on the left part (blue bar) time T0 for lipidome state before occluder therapy, while the down x-axis is differentiating the patients (2–56). The right x-axis is illustrating lipidome levels for the same patients (2.2–56.2) 6 months after undergoing therapy at time T1. Y-axis is showing the single metabolites which were analyzed. Subgroup (**a**) is illustrating the phosphatidylcholines, while subgroup (**b**) is showing lyso-phosphatidylcholines and subgroup (**c**) the sphingomyelins. In total 44 patients were measured for lipidome changes.

Six lipids did not pass the 80% LOD rule (PC aa C26:0, PC aa C30:2, PC aa C40:1, PC ae C42:0, Lyso PC a 14:0, SM C22:3). Resuming 99 metabolites of the lipidome were measured at time T0 and T1. The hierarchical cluster analysis illustrates the results for each tested metabolite subdivided in three lipid subclasses PCs, lysoPCs and SMs (Fig. 1a–c). Several metabolites of each class either in- or decreased at mid-term follow-up.

**Figure 2:**
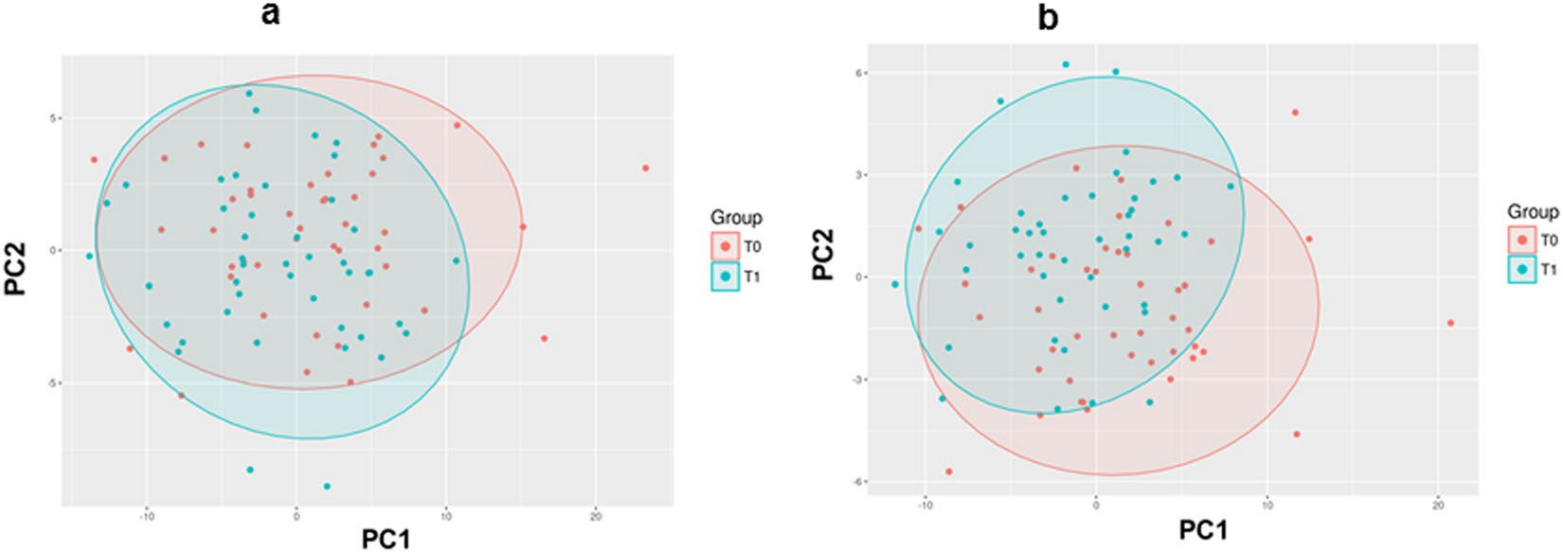
Graphical illustration of the subsumption of the multidimensional data by using a principal component analysis (**a**) and partial least squares discriminant analysis (**b**). One point per patient and group (time T0 = red points, time T1 = blue points) are subsuming the data on 2 principal components (PC1, PC2).

**Figure 3:**
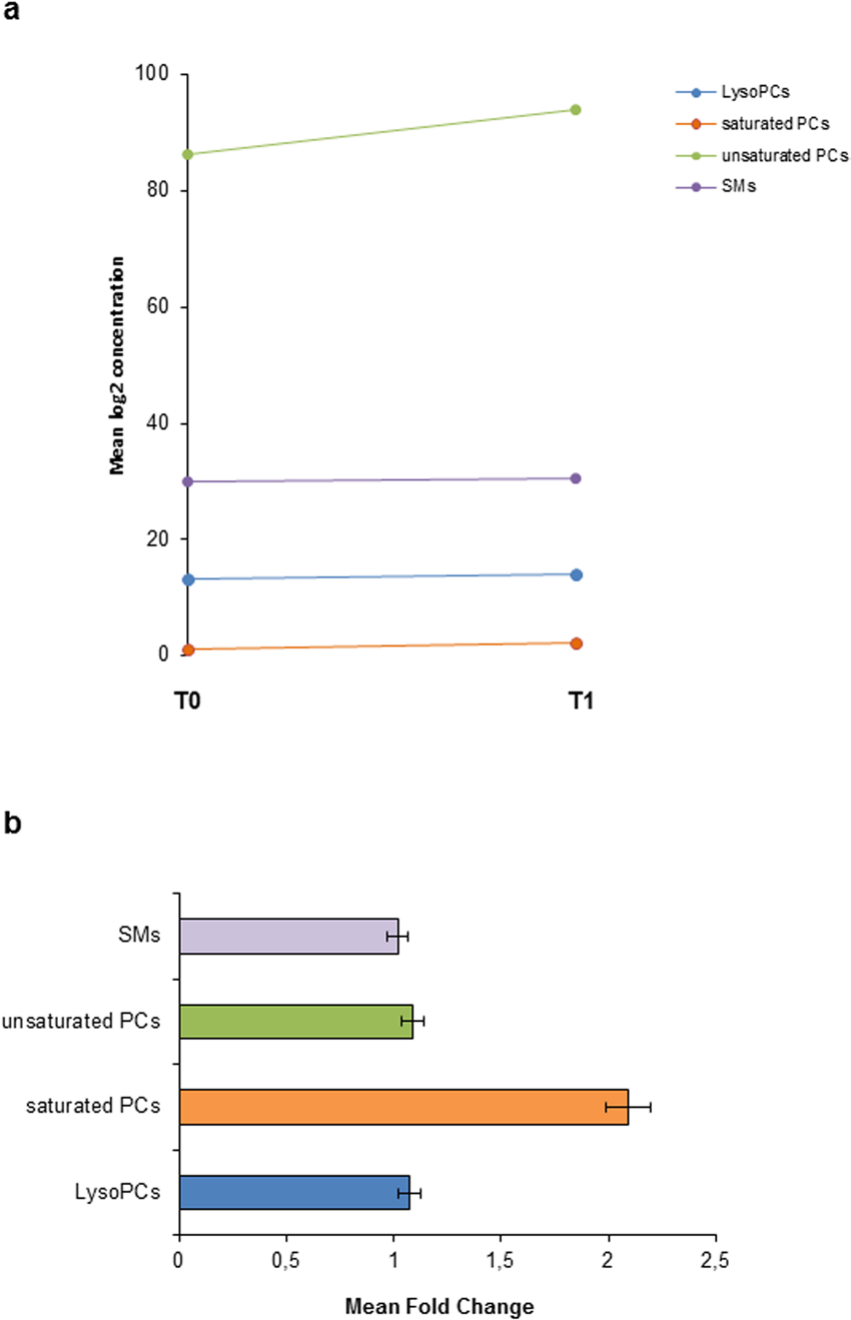
Graphic illustration of the mean concentration changes and the mean fold change at time T0 compared with T1. Metabolites were separately listed in subgroups with distinct line and bar colors (blue = lysoPCs, orange = saturated PCs, green = unsaturated PCs, purple = SMs). Significance is not considered at this summation. In total there is an increase in all 4 subclasses detectable. (**a**) Longitudinal graphic (sums of lipid classes) as mean log2 concentration. (**b**) Mean fold change diagram.

Principle component analysis (Fig. 2a) and partial least squares discriminant analysis (Fig. 2b) showed discrimination of plasma metabolome between T0 and T1. Figure 3 shows overall changes during mid-term follow-up based on lipid subclasses as mean log2 concentration. All three subclasses LysoPCs, PCs and SMs showed increases over time.

### Overall changes of lipidome metabolites before and at mid-term follow-up after successful LAAC intervention

A total of 29 PCs, 4 SMs, and 1 LysoPC significantly changed over mid-term follow-up after successful LAAC (bold typed, Table 2).

**Table 2:** Repeated measures ANOVA of the metabolite concentrations for all metabolites grouped by time “T0 vs. T1”.

Rank	Metabolite	T0		T1		p-value	Fold Change
		Mean conc. [µM]	SD	Mean conc. [µM]	SD		
1	**PC.aa.C30.0**	2.56	0.76	3.21	1.05	**<0.001**	−1.25
2	**PC.aa.C32.2**	1.58	0.79	2.20	0.96	**<0.001**	−1.39
3	**PC.aa.C32.3**	0.29	0.08	0.33	0.09	**<0.001**	−1.13
4	**PC.aa.C34.3**	10.47	3.83	12.52	4.73	**<0.001**	−1.2
5	**PC.aa.C34.4**	1.01	0.46	1.29	0.51	**<0.001**	−1.28
6	**PC.aa.C36.2**	130.08	37.94	148.25	33.26	**<0.001**	−1.14
7	**PC.ae.C40.1**	0.82	0.28	0.95	0.25	**<0.001**	−1.15
8	**PC.ae.C30.0**	0.39	0.09	0.44	0.12	**0.0012**	−1.14
9	**PC.ae.C34.2**	6.98	2.37	7.65	1.79	**0.0018**	−1.1
10	**PC.aa.C28.1**	2.28	0.66	2.62	0.78	**0.0022**	−1.15
11	**PC.ae.C36.3**	4.93	1.64	5.49	1.23	**0.0025**	−1.11
12	**PC.aa.C34.2**	263.23	73.09	289.27	67.07	**0.0036**	−1.1
13	**SM.C18.1**	9.01	3.38	8.35	3.21	**0.0049**	1.08
14	**PC.ae.C38.2**	1.33	0.38	1.50	0.35	**0.0050**	−1.13
15	**PC.ae.C38.1**	0.87	0.31	0.98	0.26	**0.0051**	−1.13
16	**PC.aa.C36.6**	0.51	0.23	0.58	0.23	**0.0053**	−1.14
17	**PC.ae.C34.3**	3.99	1.74	4.47	1.62	**0.0072**	−1.12
18	**SM..OH..C24.1**	0.86	0.27	0.95	0.22	**0.0080**	−1.1
19	**PC.aa.C40.3**	0.35	0.09	0.39	0.08	**0.0105**	−1.11
20	**PC.aa.C36.3**	76.12	23.62	85.12	21.59	**0.0113**	−1.12
21	**PC.aa.C36.1**	31.52	9.51	35.67	10.52	**0.0122**	−1.13
22	**PC.aa.C42.2**	0.17	0.04	0.19	0.04	**0.0139**	−1.11
23	**PC.aa.C24.0**	0.09	0.05	0.11	0.04	**0.0181**	−1.12
24	**PC.aa.C40.4**	2.65	0.75	2.99	0.91	**0.0189**	−1.13
25	**PC.ae.C40.3**	0.93	0.22	1.00	0.18	**0.0248**	−1.07
26	**PC.ae.C42.4**	0.57	0.14	0.62	0.15	**0.0255**	−1.09
27	**PC.ae.C30.2**	0.11	0.04	0.12	0.03	**0.0264**	−1.08
28	**PC.aa.C40.2**	0.26	0.07	0.28	0.06	**0.0265**	−1.09
29	**PC.ae.C36.4**	12.15	3.84	13.02	3.28	**0.0277**	−1.07
30	**lysoPC.a.C16.1**	2.20	0.87	2.50	0.97	**0.0287**	−1.14
31	**PC.aa.C38.3**	33.04	10.15	36.22	9.27	**0.0352**	−1.1
32	**PC.ae.C42.3**	0.49	0.12	0.53	0.11	**0.0404**	−1.08
33	**SM.C24.0**	13.06	3.27	14.40	3.97	**0.0439**	−1.1
34	**SM..OH..C22.1**	7.75	2.20	8.39	2.41	**0.0484**	−1.08
35	PC.aa.C42.4	0.15	0.03	0.16	0.03	0.0509	−1.08
36	PC.aa.C32.1	15.13	8.52	17.88	12.03	0.0535	−1.18
37	PC.aa.C32.0	11.66	2.74	12.45	2.94	0.0540	−1.07
38	PC.ae.C44.4	0.31	0.07	0.33	0.08	0.0602	−1.08
39	PC.ae.C42.1	0.31	0.07	0.33	0.07	0.0617	−1.07
40	PC.ae.C34.1	8.12	1.76	8.57	1.85	0.0758	−1.06
41	PC.ae.C34.0	1.28	0.29	1.38	0.34	0.0777	−1.07
42	PC.ae.C36.2	9.77	2.71	10.30	2.54	0.0808	−1.05
43	PC.aa.C36.4	154.50	47.59	162.58	46.91	0.0891	−1.05
44	PC.aa.C36.0	1.70	0.52	1.82	0.61	0.0988	−1.07
45	SM..OH..C14.1	5.97	1.63	6.32	1.78	0.1076	−1.06
46	PC.ae.C36.5	8.11	2.71	8.67	3.06	0.1101	−1.07
47	PC.aa.C38.4	81.35	22.62	86.14	23.64	0.1167	−1.06
48	SM.C18.0	20.56	6.21	19.50	5.53	0.1200	1.05
49	PC.ae.C38.0	1.54	0.51	1.62	0.45	0.1285	−1.05
50	PC.ae.C42.2	0.40	0.11	0.43	0.10	0.1319	−1.06
51	PC.ae.C38.3	3.02	0.73	3.20	0.72	0.1319	−1.06
52	PC.ae.C40.4	1.85	0.40	1.94	0.39	0.1332	−1.05
53	lysoPC.a.C24.0	0.19	0.07	0.21	0.06	0.1367	−1.08
54	lysoPC.a.C28.1	0.52	0.20	0.56	0.19	0.1451	−1.07
55	PC.ae.C32.1	2.09	0.57	2.21	0.65	0.1476	−1.06
56	lysoPC.a.C18.2	16.45	6.54	19.00	7.97	0.1476	−1.15
57	PC.ae.C32.2	0.62	0.18	0.65	0.17	0.1627	−1.04
58	lysoPC.a.C18.0	14.98	5.04	15.92	4.55	0.1628	−1.06
59	lysoPC.a.C16.0	67.39	20.84	71.35	18.72	0.1698	−1.06
60	SM.C16.0	103.03	20.18	107.96	23.76	0.1737	−1.05
61	PC.ae.C36.1	5.67	1.29	5.91	1.29	0.1812	−1.04
62	lysoPC.a.C20.4	5.42	2.26	5.01	2.22	0.1871	1.08
63	PC.ae.C36.0	1.19	0.35	1.30	0.54	0.2001	−1.09
64	SM.C16.1	14.66	4.30	15.28	4.53	0.2029	−1.04
65	PC.ae.C38.5	14.08	3.36	14.63	3.26	0.2055	−1.04
66	PC.aa.C42.6	0.31	0.08	0.32	0.07	0.2122	−1.05
67	SM.C26.0	0.15	0.07	0.16	0.05	0.2123	−1.03
68	PC.aa.C38.1	0.76	0.27	0.80	0.28	0.2196	−1.06
69	PC.aa.C42.5	0.28	0.08	0.29	0.06	0.2321	−1.04
70	PC.ae.C44.3	0.10	0.02	0.10	0.02	0.2362	−1.06
71	PC.aa.C40.5	7.60	1.94	8.08	2.21	0.2482	−1.06
72	PC.ae.C30.1	0.09	0.07	0.10	0.07	0.2809	−1.13
73	PC.ae.C38.6	5.04	1.52	5.22	1.52	0.2892	−1.04
74	PC.ae.C38.4	9.15	2.32	9.41	2.14	0.3103	−1.03
75	PC.aa.C36.5	19.76	11.21	20.51	9.92	0.3291	−1.04
76	SM..OH..C22.2	7.54	2.14	7.76	2.18	0.3473	−1.03
77	PC.aa.C38.5	37.71	10.56	39.05	10.16	0.3605	−1.04
78	lysoPC.a.C28.0	0.41	0.17	0.43	0.15	0.3642	−1.03
79	PC.ae.C44.5	1.56	0.39	1.63	0.49	0.4589	−1.04
80	PC.aa.C38.6	52.87	19.19	50.18	13.65	0.4660	1.05
81	lysoPC.a.C26.0	0.39	0.22	0.40	0.18	0.4685	−1.03
82	PC.aa.C42.1	0.22	0.05	0.23	0.06	0.4710	−1.04
83	PC.aa.C34.1	192.45	51.11	199.86	60.38	0.4845	−1.04
84	SM..OH..C16.1	2.89	0.77	2.82	0.75	0.4880	1.02
85	lysoPC.a.C26.1	0.21	0.11	0.22	0.11	0.5254	−1.04
86	PC.aa.C38.0	2.03	0.56	2.07	0.53	0.5380	−1.02
87	SM.C24.1	42.24	9.26	41.88	10.95	0.5866	1.01
88	PC.ae.C44.6	0.90	0.22	0.93	0.26	0.6627	−1.03
89	PC.aa.C40.6	20.13	7.34	19.94	4.83	0.6753	1.01
90	SM.C20.2	0.33	0.10	0.33	0.11	0.7009	1.01
91	SM.C26.1	0.30	0.11	0.31	0.10	0.7017	−1.01
92	PC.ae.C40.6	3.13	0.85	3.07	0.67	0.7805	1.02
93	lysoPC.a.C18.1	15.67	4.97	16.26	5.59	0.8033	−1.04
94	lysoPC.a.C17.0	1.40	0.51	1.41	0.49	0.8191	−1
95	lysoPC.a.C20.3	1.78	0.67	1.76	0.70	0.8405	1.01
96	PC.ae.C40.2	1.36	0.39	1.34	0.32	0.8746	1.01
97	PC.ae.C40.5	2.50	0.50	2.51	0.51	0.9134	−1
98	PC.ae.C42.5	1.89	0.43	1.89	0.45	0.9348	−1
99	PC.aa.C42.0	0.40	0.10	0.40	0.10	0.9587	−1

Data are presented as mean concentration [µM] ± standard deviation.

### Subgroup analyses

Lipidome metabolites were further tested in the subgroups of gender, BMI (>25 kg/m^2^ vs. <25 kg/m^2^), age (>77 years vs. <77 years), DM, LVEF (normal vs reduced <55%), creatinine (>1.2 mg/dl vs. <1.2 mg/d) and NT-proBNP (>1038 ng/l vs. <1038 ng/l). These data are given in supplementary Table 1A–G. All of the named subgroups revealed significant influence on certain metabolites in the analyzed cohort as listed in supplemental tables.

### Multivariable regression model to evaluate the influence of LAAC on lipidome

The predefined subgroups including gender, BMI, age, DM, LVEF, creatinine and NT-proBNP were adjusted within a multivariable regression model. Even after adjustment, only marginal differences in significant expression of lipidome metabolites were demonstrated (Table 3). In multivariable regression models, only follow-up time T0 vs. T1 revealed to be a significant influencing factor on lipid metabolism, whereas none of the subgroups themselves revealed any significant influence after adjustment.

**Table 3:** Results for all metabolites after a multivariate regression considering time, gender, age, DM, BMI, LVEF, creatinine and NT-proBNP. Metabolites are sorted by p-value.

Rank	Metabolite	p-value	FDR	Beta	Std. Error	t-value
1	**PC.aa.C34.4**	**<0.001**	**<**0.001	0.395	0.074	5.329
2	**PC.aa.C32.2**	**<0.001**	**<**0.001	0.532	0.101	5.250
3	**PC.aa.C30.0**	**<0.001**	0.001	0.318	0.069	4.595
4	**PC.aa.C36.2**	**<0.001**	0.010	0.211	0.055	3.826
5	**PC.aa.C32.3**	**<0.001**	0.010	0.184	0.050	3.709
6	**PC.ae.C40.1**	**<0.001**	0.010	0.238	0.064	3.690
7	**PC.aa.C34.3**	**<0.001**	0.011	0.254	0.070	3.614
8	**PC.ae.C30.0**	**0.001**	0.015	0.190	0.055	3.469
9	**PC.ae.C34.2**	**0.002**	0.019	0.174	0.052	3.337
10	**PC.aa.C28.1**	**0.002**	0.022	0.206	0.063	3.261
11	**PC.ae.C36.3**	**0.003**	0.023	0.192	0.060	3.207
12	**PC.aa.C34.2**	**0.004**	0.029	0.150	0.049	3.083
13	**SM.C18.1**	**0.005**	0.033	−0.118	0.040	−2.968
14	**PC.ae.C38.2**	**0.005**	0.033	0.198	0.067	2.957
15	**PC.ae.C38.1**	**0.005**	0.033	0.197	0.067	2.950
16	**PC.aa.C36.6**	**0.005**	0.033	0.241	0.082	2.938
17	**PC.ae.C34.3**	**0.007**	0.042	0.195	0.069	2.819
18	**SM..OH..C24.1**	**0.008**	0.044	0.167	0.060	2.780
19	**PC.aa.C40.3**	**0.011**	0.055	0.153	0.057	2.675
20	**PC.aa.C36.3**	**0.011**	0.056	0.177	0.067	2.647
21	**PC.aa.C36.1**	**0.012**	0.058	0.177	0.068	2.616
22	**PC.aa.C42.2**	**0.014**	0.062	0.151	0.059	2.566
23	**PC.aa.C24.0**	**0.018**	0.078	0.208	0.085	2.458
24	**PC.aa.C40.4**	**0.019**	0.078	0.170	0.069	2.440
25	**PC.ae.C40.3**	**0.025**	0.094	0.108	0.047	2.326
26	**PC.ae.C42.4**	**0.025**	0.094	0.124	0.054	2.314
27	**PC.ae.C30.2**	**0.026**	0.094	0.140	0.061	2.300
28	**PC.aa.C40.2**	**0.027**	0.094	0.149	0.065	2.297
29	**PC.ae.C36.4**	**0.028**	0.095	0.120	0.053	2.279
30	**lysoPC.a.C16.1**	**0.029**	0.095	0.184	0.081	2.264
31	**PC.aa.C38.3**	**0.035**	0.112	0.148	0.068	2.175
32	**PC.ae.C42.3**	**0.040**	0.125	0.119	0.056	2.113
33	**SM.C24.0**	**0.044**	0.132	0.136	0.066	2.076
34	**SM..OH..C22.1**	**0.048**	0.141	0.114	0.056	2.031
35	PC.aa.C42.4	0.051	0.144	0.127	0.063	2.008
36	PC.aa.C32.1	0.054	0.145	0.180	0.090	1.985
37	PC.aa.C32.0	0.054	0.145	0.091	0.046	1.981
38	PC.ae.C44.4	0.060	0.157	0.110	0.057	1.930
39	PC.ae.C42.1	0.062	0.157	0.104	0.054	1.919
40	PC.ae.C34.1	0.076	0.188	0.079	0.044	1.820
41	PC.ae.C34.0	0.078	0.188	0.097	0.054	1.807
42	PC.ae.C36.2	0.081	0.190	0.093	0.052	1.788
43	PC.aa.C36.4	0.089	0.205	0.078	0.045	1.739
44	PC.aa.C36.0	0.099	0.222	0.104	0.062	1.687
45	SM..OH..C14.1	0.108	0.237	0.084	0.051	1.643
46	PC.ae.C36.5	0.110	0.237	0.095	0.058	1.631
47	PC.aa.C38.4	0.117	0.246	0.085	0.053	1.601
48	SM.C18.0	0.120	0.248	0.072	0.045	−1.586
49	PC.ae.C38.0	0.129	0.254	0.106	0.068	1.550
50	PC.ae.C42.2	0.132	0.254	0.102	0.066	1.536
51	PC.ae.C38.3	0.132	0.254	0.094	0.061	1.536
52	PC.ae.C40.4	0.133	0.254	0.071	0.046	1.530
53	lysoPC.a.C24.0	0.137	0.255	0.136	0.090	1.516
54	lysoPC.a.C28.1	0.145	0.261	0.115	0.078	1.484
55	PC.ae.C32.1	0.148	0.261	0.076	0.052	1.475
56	lysoPC.a.C18.2	0.148	0.261	0.174	0.118	1.475
57	PC.ae.C32.2	0.163	0.278	0.071	0.050	1.420
58	lysoPC.a.C18.0	0.163	0.278	0.115	0.081	1.420
59	lysoPC.a.C16.0	0.170	0.285	0.105	0.075	1.396
60	SM.C16.0	0.174	0.287	0.062	0.045	1.383
61	PC.ae.C36.1	0.181	0.294	0.068	0.050	1.359
62	lysoPC.a.C20.4	0.187	0.299	−0.125	0.093	−1.341
63	PC.ae.C36.0	0.200	0.313	0.090	0.069	1.301
64	SM.C16.1	0.203	0.313	0.054	0.041	1.293
65	PC.ae.C38.5	0.206	0.313	0.061	0.047	1.285
66	PC.aa.C42.6	0.212	0.314	0.079	0.062	1.266
67	SM.C26.0	0.212	0.314	0.099	0.078	1.266
68	PC.aa.C38.1	0.220	0.320	0.098	0.078	1.246
69	PC.aa.C42.5	0.232	0.333	0.076	0.063	1.212
70	PC.ae.C44.3	0.236	0.334	0.072	0.060	1.201
71	PC.aa.C40.5	0.248	0.346	0.082	0.070	1.171
72	PC.ae.C30.1	0.281	0.386	0.221	0.202	1.092
73	PC.ae.C38.6	0.289	0.392	0.060	0.056	1.073
74	PC.ae.C38.4	0.310	0.415	0.048	0.047	1.027
75	PC.aa.C36.5	0.329	0.434	0.099	0.100	0.987
76	SM..OH..C22.2	0.347	0.452	0.040	0.042	0.950
77	PC.aa.C38.5	0.361	0.462	0.055	0.059	0.924
78	lysoPC.a.C28.0	0.364	0.462	0.079	0.086	0.917
79	PC.ae.C44.5	0.459	0.569	0.041	0.055	0.747
80	PC.aa.C38.6	0.466	0.569	−0.044	0.060	−0.736
81	lysoPC.a.C26.0	0.468	0.569	0.080	0.110	0.731
82	PC.aa.C42.1	0.471	0.569	0.042	0.057	0.727
83	PC.aa.C34.1	0.484	0.575	0.036	0.051	0.705
84	SM..OH..C16.1	0.488	0.575	−0.033	0.048	−0.699
85	lysoPC.a.C26.1	0.525	0.612	0.063	0.098	0.640
86	PC.aa.C38.0	0.538	0.619	0.031	0.050	0.621
87	SM.C24.1	0.587	0.667	−0.026	0.048	−0.548
88	PC.ae.C44.6	0.663	0.746	0.023	0.053	0.439
89	PC.aa.C40.6	0.675	0.751	0.027	0.064	0.422
90	SM.C20.2	0.701	0.763	−0.023	0.061	−2.219
91	SM.C26.1	0.702	0.763	0.026	0.068	0.386
92	PC.ae.C40.6	0.781	0.840	−0.014	0.051	−0.280
93	lysoPC.a.C18.1	0.803	0.855	0.024	0.096	0.251
94	lysoPC.a.C17.0	0.819	0.863	0.023	0.100	0.230
95	lysoPC.a.C20.3	0.840	0.876	−0.022	0.106	−0.202
96	PC.ae.C40.2	0.875	0.902	−0.008	0.049	−0.159
97	PC.ae.C40.5	0.913	0.932	0.005	0.045	0.109
98	PC.ae.C42.5	0.935	0.944	−0.004	0.043	−0.082
99	PC.aa.C42.0	0.959	0.959	0.003	0.056	0.052

FDR = False-Discovery-Rate.

### Graphical visualization of lipid metabolites being influenced by LAAC

**Figure 4:**
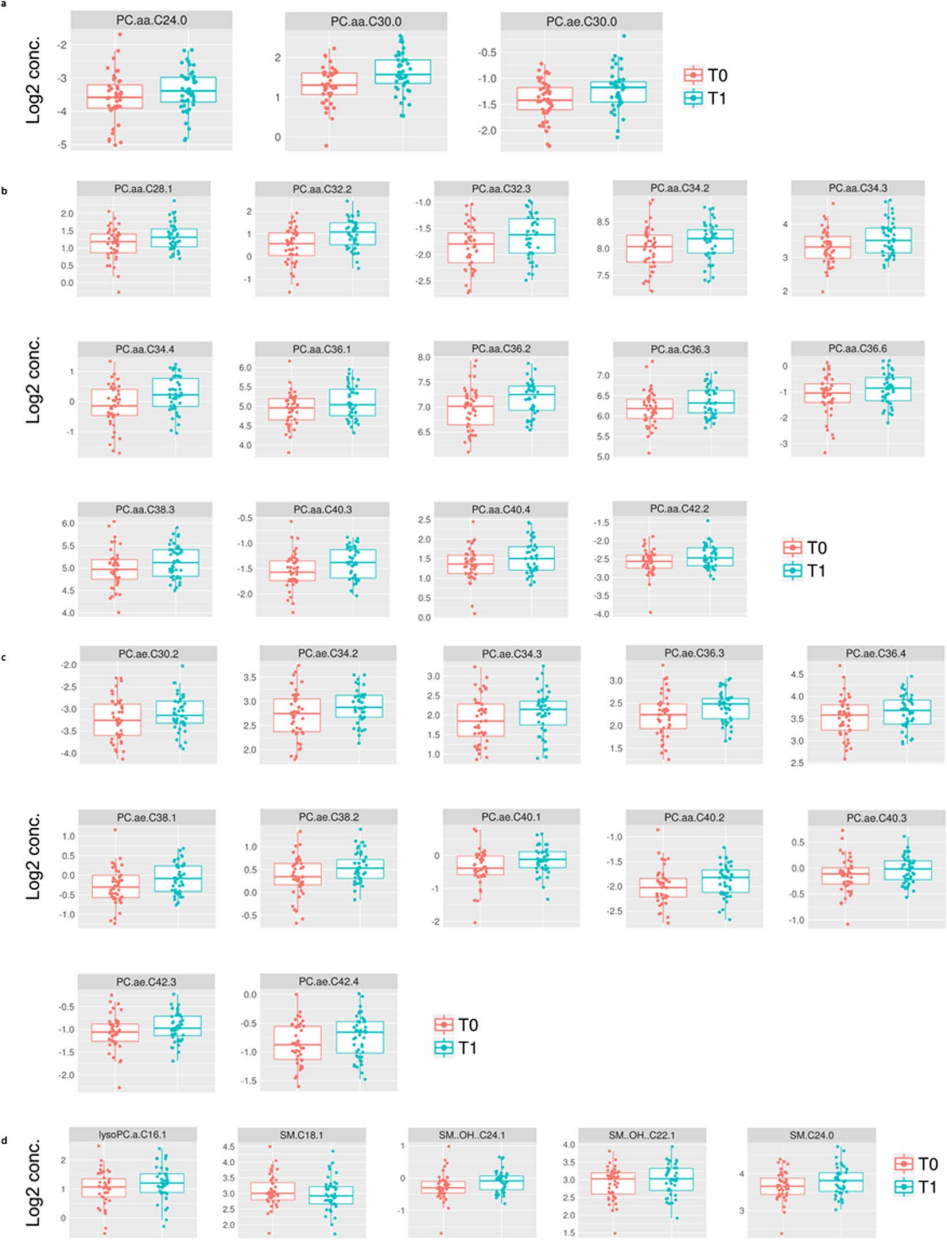
Illustration of all metabolites with a significant concentration change as log2 concentration (y-axis). Each dot is showing the result of one patient with in total 44 dots representing all included patient-samples. (**a**) saturated Phosphatidylcholines (**b**) Unsaturated diacyl-Phosphatidylcholines (**c**) Unsaturated acyl-alkyl-Phosphatidylcholines (**d**) lysoPC.a.C16.1. and SMs.

**Figure 5:**
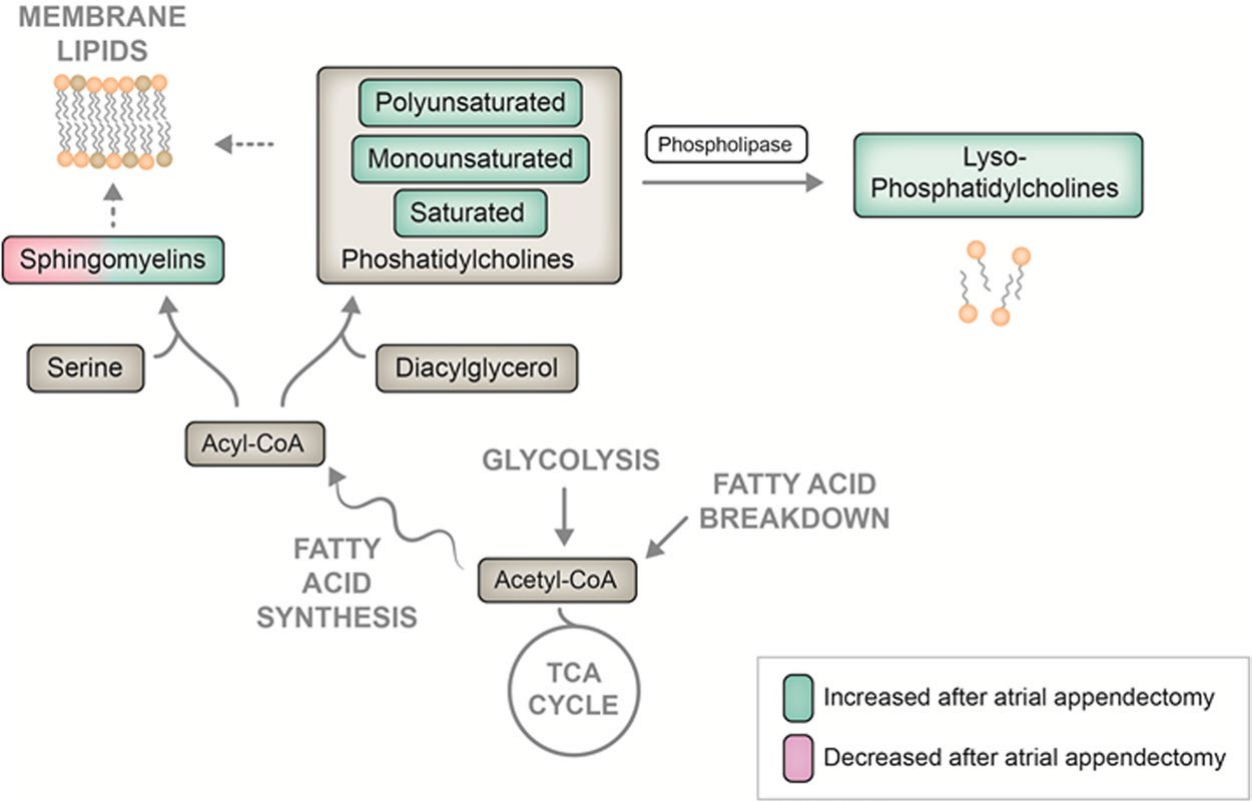
A schematic overview of intra-cellular lipid metabolism and an illustration of the results. While SMs have shown a distinct serum level behavior with increasing and decreasing levels, the other subclasses are strictly increased after LAAC therapy.

The distribution of each significant metabolomic (after multivariable adjustment) is visualized in Fig. 4. Overall 33 metabolites increased significantly, whereas only one metabolite decreased significantly over time (i.e. SM.C18.1). Figure 5 illustrates the intracellular lipid pathways implementing the final significant changes.

## Discussion

The present study demonstrates for the first time that successful LAAC significantly impacts lipid metabolism within mid-term follow-up. It was demonstrated that only time after successful LAAC (i.e. mid-term follow-up period) revealed to be associated significantly on the alterations of lipid metabolites, which might indirectly reflect the effect of successful LAAC, as the main cause for these differences. The subgroups within our open-label study cohort did not reveal a significant influence, which makes biases by patient characteristics unlikely.

### General hypotheses

Interactions between cardiovascular diseases and the plasma lipidome were shown to reveal both cardio-protective and -toxic effects^18–20^. On the other hand, the tissue of the LAA, respectively LAA cardiomyocytes, are supposed to reveal different physiological and histological characteristics compared to left atrial tissue itself. The LAA cardiomyocytes form comb-like trabeculae within the appendage, which combined with relative blood stasis in AF predestinates the LAA for thrombus formation and increases the risk for stroke in patients with AF^21^. Furthermore, in AF the LAA is undergoing remodeling processes affecting its role as an endocrine organ^7,8,22^.

Lipid metabolites represent a main source of energy supply for human cells. Depending on their biochemical properties, lipids are subdivided traditionally into the main sub-classes like free fatty acids (FFA), triglycerides (TG), phospholipids (PL) or sphingolipids (SL)^23^. Furthermore, subsequent underclasses consist of saturated or unsaturated lipids. Their lipophilic structure necessitates a protein-linked transport in blood plasma. Pathological alterations of the reference plasma lipoproteins such as high density (HDL), low density lipoproteins (LDL) or chylomicrons contribute as established cardiovascular risk factors causing hyper- or dyslipidemias and are associated with an adverse cardiovascular outcome^24^.

Some lipid sub-classes like PLs being measured in this study are essential structural components of the cell membrane. PLs are commonly defined as phosphorylated lipids and mainly consist of glycerophospholipid (gPL) and SL species. Structurally, SLs are composed of a long-chain sphingoid base, an amide-linked fatty acid, and a polar head group at the 1-position. Phosphorylated SLs are called SMs and therefore part of the PL subclass^25^. gPLs on the other hand consist of a glycerol based structure esterified with two varying organic fatty acids and one molecule of phosphoric acid. gPL species like phosphatidylcholine (PC), phosphatidylethanolamine (PE) or phosphatidylserine (PS) can be synthetized through attached various alcohols^26,27^. Lipase enzymes in turn can hydrolyze gPLs and form corresponding lyso-phospholipids like lysoPCs^28^.

### Effects on intra-cellular lipid metabolism

After undergoing intra-cellular lipolysis, PLs and TGs develop so called free fatty acids (FFAs), which serve as carrier molecules for acetyl-CoA, the main molecule within the citric acid cycle (TCA). Intermediate metabolites from TCA in turn are mainly used within the oxidative phosphorylation pathway at the inner mitochondrial membrane finally generating adenosine triphosphate (ATP) - the main cellular source of energy. On the other hand, TCA may deliver acyl-CoA, which can be used to as a substrate in fatty acid synthesis. Fatty acids can further be processed to PLs, SM and LysoPLs, as being outlined in the present analysis (Fig. 5). The present data show an increase of serum lipid subclasses lysoPCs, PCs and partially SMs, which might be interpreted as a hint for a developing imbalance of lipid metabolism with a predominating synthesis of fatty acids and subsequent lipid-subclasses after successful LAAC. Reduced fatty acid oxidation has already been associated with the development of cardiac hypertrophy^29^.

### Effects on sphingomyelins

The SMs SM..OH..C24.1, SM.C24.0 and SM..OH..C22.1 have shown an increase, whereas SM.C18.1 was solely decreased significantly. Within the SLs several metabolites are known to be bioactive molecules regulating signal transduction pathways and influence on cell-cell interactions^30^. Focusing on cardiovascular diseases, ceramide – the basic SL - was shown to be involved in cardiac ischemia and reperfusion injury. The exact pathophysiology is not yet clarified, but ceramide seems to mediate apoptosis via activation of CD95 and tumor necrosis factor receptors resulting in cardio-toxic effects^18,31,32^. Besides a denovo synthesis pathway ceramide can be produced directly through SMs^30^. Decreased levels of SM.C18.1 and increased levels of the other SMs might potentially reflect an imbalance in ceramide synthesis. Altered forms of bioactive SL were shown in cardiovascular diseases such as myocardial infarction^33,34^.

### Effects on phosphatidylcholines and lysophosphatidylcholines

A total of 29 PCs with attaching mostly higher carbon numbers fatty acids were significantly increased after successful LAAC.

As known, hydrolyzed PCs do form corresponding lysoPCs^28^, and several lysoPCs were measured in the present study, revealing LysoPC.a.C16.1 being increased over time. Furthermore, increased levels of several PCs constitute a higher amount of substrates for the later synthesis of lysoPCs. Balances between both sub-classes represent important intra-cellular pathways, because lysoPCs are known to reveal pro-inflammatory and atherogenic effects^19^. While there are no direct cardio-modulating effects of PCs described so far, increased levels of PCs from gut bacteria such as trimethylamine N-oxide (TMAO) and choline may be associated with an increased cardiovascular risk profile^35–37^. Choline was shown to be associated with the future development of adverse cardiac events in addition to troponins^38^.

## Conclusions

Several metabolites from lipid classes of PCs, SMs and lysoPCs were altered after successful LAAC treatment in patients with non-valvular AF. It may be speculated whether these alterations might reflect potential pro-atherogenic and pro-inflammatory changes at mid-term follow-up after LAAC. Therefore, a more differentiated clinical follow-up of these patients may be widened to metabolomic alterations, and future research may show-up the pathophysiological relevance of the present findings.

### Study limitations

The present study is the first evaluating the influence of successful LAAC on lipid metabolism and is therefore descriptive and hypothesis generating. The main limitations are the small sample size, lack of a control group without LAAC, lack of a control group according to drug treatment (especially statins therapy) and the pre-defined follow-up time of mid-term 6 months, disregarding short-term or long-term effects after LAAC. A systemic increase of potential atherogenic and proinflammatory metabolites being measured in plasma must not represent necessarily local pathological processes of cardiac tissue.

## Electronic supplementary material

Supplementary Dataset 1
