# Supplementary material for: Percutaneous Closure of Left Atrial Appendage significantly affects Lipidome Metabolism
Source: Sci Rep. 2018 Apr 12;8:5894. doi: 10.1038/s41598-018-23935-w (PMC5897373; doi:10.1038/s41598-018-23935-w)
Supplement: Supplementary file 1 — Supplementary Dataset 1 [file 41598_2018_23935_MOESM1_ESM.doc]

**Percutaneous Closure of Left Atrial Appendage significantly affects Lipidome Metabolism**

Running title: Lipidome metabolism in LAAC

Yücel G. ^1,2^*, Behnes M. ^1^*, Barth C. ^1^, Wenke A. ^1^, Sartorius B. ^1^, Mashayekhi K. ^3^, Yazdani B. ^4^, Bertsch T. ^5^, Rusnak J. ^1^, Saleh A. ^1,2^, Hoffmann U. ^1^, Fastner C. ^1^, Lang S. ^1,2^, Zhou X. ^1,2^, Sattler K. ^1^, Borggrefe M. ^1,2^, Akin I. ^1,2^.

**both authors contributed equally to the study.*

From the First Department of Medicine, Faculty of Medicine, University Medical Centre Mannheim (UMM), University of Heidelberg, Mannheim, Germany

1 - First Department of Medicine, Faculty of Medicine, University Medical Centre Mannheim (UMM), University of Heidelberg, Mannheim, Germany.

2 - DZHK (German Center for Cardiovascular Research), Partner Site, Heidelberg-Mannheim, Mannheim, Germany.

3 - Clinic for Cardiology and Angiology II, Universitäts-Herzzentrum Freiburg - Bad Krozingen, Bad Krozingen, Germany.

4 - Fifth Department of Medicine, Faculty of Medicine, University Medical Centre Mannheim (UMM), University of Heidelberg, Mannheim, Germany.

5 - Institute of Clinical Chemistry, Laboratory Medicine and Transfusion Medicine, General Hospital Nuremberg and Paracelsus Medical University, Nuremberg, Germany

|  |  | **Gender** | | | | | | | |  | |  |
| --- | --- | --- | --- | --- | --- | --- | --- | --- | --- | --- | --- | --- |
|  |  | **T0** | | | | **T1** | | | |  | |  |
|  |  | **Female (N=14)** | | **Male (N=30)** | | **Female (N=14)** | | **Male (N=30)** | | **rANOVA** | | |
| **Rank** | **Metabolite** | **Mean conc. [µM]** | **SD** | **Mean conc. [µM]** | **SD** | **Mean conc. [µM]** | **SD** | **Mean conc. [µM]** | **SD** | **p-Value** | **FDR** | |
| 1 | PC.aa.C32.2 | 1.42 | 0.62 | 1.66 | 0.85 | 2.10 | 0.99 | 2.25 | 0.95 | 0.0205 | 0.3446 | |
| 2 | SM.C16.0 | 113.46 | 19.51 | 98.16 | 18.58 | 118.44 | 18.21 | 103.07 | 24.46 | 0.0266 | 0.3446 | |
| 3 | PC.aa.C40.5 | 7.70 | 1.98 | 7.55 | 1.92 | 8.40 | 2.78 | 7.93 | 1.86 | 0.0284 | 0.3446 | |
| 4 | PC.ae.C40.4 | 1.97 | 0.30 | 1.79 | 0.43 | 2.07 | 0.36 | 1.87 | 0.39 | 0.0303 | 0.3446 | |
| 5 | PC.ae.C42.3 | 0.50 | 0.10 | 0.49 | 0.13 | 0.55 | 0.10 | 0.52 | 0.12 | 0.0303 | 0.3446 | |
| 6 | PC.aa.C42.2 | 0.18 | 0.04 | 0.17 | 0.04 | 0.20 | 0.03 | 0.18 | 0.05 | 0.0315 | 0.3446 | |
| 7 | SM..OH..C24.1 | 0.94 | 0.36 | 0.83 | 0.20 | 1.03 | 0.24 | 0.92 | 0.21 | 0.0357 | 0.3446 | |
| 8 | SM.C24.0 | 13.87 | 3.35 | 12.69 | 3.16 | 15.15 | 3.57 | 14.05 | 4.10 | 0.0381 | 0.3446 | |
| 9 | PC.ae.C38.4 | 9.51 | 2.30 | 8.99 | 2.31 | 9.82 | 2.18 | 9.22 | 2.09 | 0.0458 | 0.3446 | |
| Supplemental Table 1A. Repeated measures ANOVA of the metabolite concentrations for significantly altered metabolites grouped by “Gender female vs. male” at time T0 and T1. Data are presented as mean concentration [µM] ± standard deviation. FDR, false discovery rate. P values are given for mean changes from T0 vs. T1 according to the outlined subgroup. | | | | | | | | | | | | |

|  |  | **BMI** | | | | | | | |  |  |
| --- | --- | --- | --- | --- | --- | --- | --- | --- | --- | --- | --- |
|  |  | **T0** | | | | **T1** | | | |  |  |
|  |  | **BMI >25 (N=29)** | | **BMI < 25 (N=15)** | | **BMI >25 (N=29)** | | **BMI < 25 (N=15)** | | **rANOVA** | |
| **Rank** | **Metabolite** | **Mean conc. [µM]** | **SD** | **Mean conc. [µM]** | **SD** | **Mean conc. [µM]** | **SD** | **Mean conc. [µM]** | **SD** | **p-Value** | **FDR** |
| 1 | PC.ae.C38.0 | 1.54 | 0.59 | 1.53 | 0.32 | 1.60 | 0.50 | 1.65 | 0.34 | 0.001 | 0.099 |
| 2 | PC.aa.C36.2 | 127.98 | 36.29 | 134.16 | 40.62 | 146.76 | 36.06 | 151.13 | 26.81 | 0.0083 | 0.2698 |
| 3 | PC.ae.C34.3 | 3.61 | 1.33 | 4.72 | 2.17 | 4.15 | 1.57 | 5.08 | 1.56 | 0.0108 | 0.2698 |
| 4 | PC.ae.C36.2 | 9.46 | 2.47 | 10.37 | 3.02 | 10.08 | 2.79 | 10.73 | 1.89 | 0.0165 | 0.2698 |
| 5 | PC.aa.C40.4 | 2.74 | 0.78 | 2.48 | 0.65 | 3.12 | 0.99 | 2.74 | 0.67 | 0.0216 | 0.2698 |
| 6 | lysoPC.a.C26.0 | 0.38 | 0.13 | 0.39 | 0.33 | 0.42 | 0.20 | 0.34 | 0.10 | 0.0233 | 0.2698 |
| 7 | PC.ae.C36.3 | 4.71 | 1.31 | 5.35 | 2.07 | 5.34 | 1.23 | 5.76 | 1.19 | 0.0267 | 0.2698 |
| 8 | PC.aa.C34.3 | 10.35 | 3.88 | 10.72 | 3.72 | 12.53 | 5.18 | 12.51 | 3.73 | 0.0302 | 0.2698 |
| 9 | PC.ae.C30.0 | 0.39 | 0.09 | 0.39 | 0.10 | 0.45 | 0.14 | 0.43 | 0.08 | 0.0321 | 0.2698 |
| 10 | PC.aa.C36.6 | 0.50 | 0.26 | 0.52 | 0.15 | 0.57 | 0.26 | 0.61 | 0.17 | 0.0334 | 0.2698 |
| 11 | PC.ae.C40.6 | 3.12 | 1.00 | 3.17 | 0.42 | 3.04 | 0.77 | 3.12 | 0.37 | 0.035 | 0.2698 |
| 12 | PC.ae.C40.4 | 1.86 | 0.38 | 1.83 | 0.44 | 1.95 | 0.42 | 1.91 | 0.33 | 0.0397 | 0.2698 |
| 13 | PC.aa.C32.0 | 11.47 | 2.71 | 12.03 | 2.76 | 12.33 | 3.13 | 12.67 | 2.52 | 0.041 | 0.2698 |
| 14 | SM..OH..C16.1 | 2.90 | 0.91 | 2.87 | 0.40 | 2.83 | 0.86 | 2.81 | 0.49 | 0.0413 | 0.2698 |
| 15 | PC.ae.C32.2 | 0.61 | 0.17 | 0.64 | 0.19 | 0.63 | 0.16 | 0.67 | 0.16 | 0.0436 | 0.2698 |
| 16 | PC.ae.C42.3 | 0.48 | 0.11 | 0.51 | 0.14 | 0.52 | 0.12 | 0.55 | 0.10 | 0.047 | 0.2698 |
| 17 | PC.ae.C40.1 | 0.80 | 0.28 | 0.86 | 0.28 | 0.94 | 0.28 | 0.98 | 0.18 | 0.0482 | 0.2698 |
| Supplemental Table 1B. Repeated measures ANOVA of the metabolite concentrations for significantly altered metabolites grouped by “BMI>25kg/m^2^ vs BMI<25kg/m^2^” at time T0 and T1. Data are presented as mean concentration [µM] ± standard deviation. FDR, false discovery rate. BMI, body mass index. P values are given for mean changes from T0 vs. T1 according to the outlined subgroup. | | | | | | | | | | | |

|  |  | **Age** | | | | | | | |  |  |
| --- | --- | --- | --- | --- | --- | --- | --- | --- | --- | --- | --- |
|  |  | **T0** | | | | **T1** | | | |  |  |
|  |  | **Age ≥ 77 (N=23)** | | **Age < 77 (N=21)** | | **Age ≥ 77 (N=23)** | | **Age < 77 (N=21)** | | **rANOVA** | |
| **Rank** | **Metabolite** | **Mean conc. [µM]** | **SD** | **Mean conc. [µM]** | **SD** | **Mean conc. [µM]** | **SD** | **Mean conc. [µM]** | **SD** | **p-Value** | **FDR** |
| 1 | PC.aa.C32.2 | 1.41 | 0.61 | 1.76 | 0.90 | 1.95 | 0.71 | 2.45 | 1.11 | 0.0023 | 0.2277 |
| 2 | lysoPC.a.C16.1 | 2.06 | 0.66 | 2.34 | 1.01 | 2.33 | 0.81 | 2.66 | 1.08 | 0.0087 | 0.4306 |
| 3 | PC.aa.C42.2 | 0.17 | 0.03 | 0.17 | 0.04 | 0.20 | 0.05 | 0.18 | 0.04 | 0.0314 | 0.4928 |
| 4 | PC.ae.C38.1 | 0.81 | 0.20 | 0.93 | 0.38 | 0.93 | 0.24 | 1.03 | 0.27 | 0.0315 | 0.4928 |
| 5 | PC.aa.C36.2 | 120.80 | 24.36 | 139.37 | 45.97 | 139.04 | 30.38 | 157.45 | 33.46 | 0.036 | 0.4928 |
| Supplemental Table 1C. Repeated measures ANOVA of the metabolite concentrations for significantly altered metabolites grouped by “age>77 years vs. age<77 years” at time T0 and T1. Data are presented as mean concentration [µM] ± standard deviation. FDR, false discovery rate. P values are given for mean changes from T0 vs. T1 according to the outlined subgroup. | | | | | | | | | | | |

|  |  | **Diabetes Mellitus type II** | | | | | | | |  |  |
| --- | --- | --- | --- | --- | --- | --- | --- | --- | --- | --- | --- |
|  |  | **T0** | | | | **T1** | | | |  |  |
|  |  | **DM II (N=16)** | | **No DM II (N=28)** | | **DM II (N=16)** | | **No DM II (N=28)** | | **rANOVA** | |
| **Rank** | **Metabolite** | **Mean conc. [µM]** | **SD** | **Mean conc. [µM]** | **SD** | **Mean conc. [µM]** | **SD** | **Mean conc. [µM]** | **SD** | **p-Value** | **FDR** |
| 1 | PC.ae.C34.2 | 6.31 | 1.80 | 7.37 | 2.55 | 7.02 | 1.71 | 8.01 | 1.73 | 0.0006 | 0.0594 |
| 2 | PC.ae.C40.3 | 0.93 | 0.29 | 0.93 | 0.16 | 0.99 | 0.23 | 1.00 | 0.15 | 0.0027 | 0.1337 |
| 3 | PC.ae.C34.3 | 3.27 | 1.05 | 4.40 | 1.92 | 3.90 | 1.70 | 4.79 | 1.48 | 0.0055 | 0.1815 |
| 4 | lysoPC.a.C16.1 | 2.22 | 1.19 | 2.18 | 0.61 | 2.41 | 1.05 | 2.55 | 0.91 | 0.0149 | 0.297 |
| 5 | lysoPC.a.C24.0 | 0.20 | 0.04 | 0.19 | 0.08 | 0.22 | 0.07 | 0.20 | 0.06 | 0.0154 | 0.297 |
| 6 | PC.ae.C36.3 | 4.56 | 1.35 | 5.15 | 1.74 | 5.20 | 1.41 | 5.65 | 1.09 | 0.021 | 0.297 |
| 7 | PC.ae.C30.0 | 0.40 | 0.12 | 0.38 | 0.08 | 0.45 | 0.14 | 0.44 | 0.11 | 0.0221 | 0.297 |
| 8 | PC.ae.C44.3 | 0.10 | 0.02 | 0.10 | 0.02 | 0.10 | 0.03 | 0.11 | 0.02 | 0.0295 | 0.297 |
| 9 | PC.aa.C42.0 | 0.38 | 0.11 | 0.41 | 0.09 | 0.38 | 0.10 | 0.41 | 0.09 | 0.0316 | 0.297 |
| 10 | PC.aa.C36.6 | 0.55 | 0.32 | 0.49 | 0.15 | 0.57 | 0.25 | 0.58 | 0.22 | 0.0334 | 0.297 |
| 11 | PC.aa.C34.4 | 1.02 | 0.47 | 1.01 | 0.45 | 1.29 | 0.54 | 1.30 | 0.49 | 0.0372 | 0.297 |
| 12 | PC.aa.C42.2 | 0.17 | 0.05 | 0.17 | 0.03 | 0.19 | 0.05 | 0.19 | 0.04 | 0.039 | 0.297 |
| 13 | PC.ae.C38.5 | 14.13 | 3.42 | 14.06 | 3.33 | 14.75 | 4.00 | 14.56 | 2.74 | 0.039 | 0.297 |
| 14 | PC.ae.C38.0 | 1.58 | 0.67 | 1.51 | 0.39 | 1.60 | 0.50 | 1.63 | 0.42 | 0.042 | 0.297 |
| 15 | PC.ae.C36.0 | 1.30 | 0.39 | 1.12 | 0.31 | 1.41 | 0.61 | 1.23 | 0.49 | 0.0481 | 0.3175 |
| Supplemental Table 1D. Repeated measures ANOVA of the metabolite concentrations for significantly altered metabolites grouped by “Diabetes mellitus type II yes vs. no” at time T0 and T1. Data are presented as mean concentration [µM] ± standard deviation. FDR, false discovery rate. DM II, diabetes mellitus type II. P values are given for mean changes from T0 vs. T1 according to the outlined subgroup. | | | | | | | | | | | |

|  |  | **LVEF** | | | | | | | |  |  |
| --- | --- | --- | --- | --- | --- | --- | --- | --- | --- | --- | --- |
|  |  | **T0** | | | | **T1** | | | |  |  |
|  |  | **Normal LVEF (N=34)** | | **Reduced LVEF (N=10)** | | **Normal LVEF (N=34)** | | **Reduced LVEF (N=10)** | | **rANOVA** | |
| **Rank** | **Metabolite** | **Mean conc. [µM]** | **SD** | **Mean conc. [µM]** | **SD** | **Mean conc. [µM]** | **SD** | **Mean conc. [µM]** | **SD** | **p-Value** | **FDR** |
| 1 | PC.aa.C32.2 | 1.63 | 0.79 | 1.43 | 0.75 | 2.29 | 1.02 | 1.89 | 0.66 | 0.0086 | 0.6138 |
| 2 | PC.aa.C34.4 | 1.01 | 0.46 | 1.01 | 0.47 | 1.29 | 0.50 | 1.29 | 0.53 | 0.0124 | 0.6138 |
| Supplemental Table 1E. Repeated measures ANOVA of the metabolite concentrations for significantly altered metabolites grouped by “Normal vs. reduced LVEF” at time T0 and T1. Normal LVEF was defined as > 55%. Data are presented as mean concentration [µM] ± standard deviation. FDR, false discovery rate. LVEF, left ventricular ejection fraction. P values are given for mean changes from T0 vs. T1 according to the outlined subgroup. | | | | | | | | | | | |

|  |  | **Creatinine** | | | | | | | |  |  |
| --- | --- | --- | --- | --- | --- | --- | --- | --- | --- | --- | --- |
|  |  | **T0** | | | | **T1** | | | |  |  |
|  |  | **Crea ≥ 1.2mg/dl (N=14)** | | **Crea< 1.2mg/dl (N=30)** | | **Crea ≥ 1.2mg/dl (N=14)** | | **Crea < 1.2mg/dl (N=30)** | | **rANOVA** | |
| **Rank** | **Metabolite** | **Mean conc. [µM]** | **SD** | **Mean conc. [µM]** | **SD** | **Mean conc. [µM]** | **SD** | **Mean conc. [µM]** | **SD** | **p-Value** | **FDR** |
| 1 | PC.ae.C36.0 | 1.30 | 0.38 | 1.13 | 0.32 | 1.25 | 0.41 | 1.32 | 0.60 | 0.0011 | 0.1089 |
| 2 | PC.ae.C44.3 | 0.09 | 0.01 | 0.10 | 0.02 | 0.10 | 0.03 | 0.11 | 0.02 | 0.0034 | 0.1287 |
| 3 | PC.ae.C38.1 | 0.85 | 0.24 | 0.88 | 0.33 | 0.93 | 0.25 | 1.00 | 0.26 | 0.0045 | 0.1287 |
| 4 | PC.aa.C38.3 | 31.73 | 12.37 | 33.65 | 8.85 | 34.92 | 11.02 | 36.83 | 8.26 | 0.0052 | 0.1287 |
| 5 | PC.ae.C42.2 | 0.38 | 0.12 | 0.41 | 0.10 | 0.37 | 0.10 | 0.45 | 0.09 | 0.0109 | 0.2158 |
| 6 | PC.ae.C38.2 | 1.32 | 0.31 | 1.33 | 0.40 | 1.41 | 0.32 | 1.54 | 0.35 | 0.0147 | 0.2426 |
| 7 | PC.ae.C42.1 | 0.28 | 0.08 | 0.32 | 0.07 | 0.30 | 0.07 | 0.34 | 0.06 | 0.0199 | 0.2661 |
| 8 | SM..OH..C22.1 | 7.01 | 2.57 | 8.10 | 1.91 | 7.80 | 2.96 | 8.66 | 2.04 | 0.0215 | 0.2661 |
| 9 | PC.aa.C40.3 | 0.34 | 0.11 | 0.36 | 0.07 | 0.39 | 0.10 | 0.39 | 0.07 | 0.0273 | 0.2856 |
| 10 | PC.ae.C32.2 | 0.62 | 0.20 | 0.62 | 0.17 | 0.62 | 0.17 | 0.66 | 0.16 | 0.0322 | 0.2856 |
| 11 | PC.aa.C42.2 | 0.17 | 0.05 | 0.17 | 0.03 | 0.18 | 0.06 | 0.19 | 0.03 | 0.0345 | 0.2856 |
| 12 | PC.ae.C36.5 | 8.48 | 3.07 | 7.94 | 2.50 | 8.73 | 4.01 | 8.65 | 2.48 | 0.0351 | 0.2856 |
| 13 | PC.aa.C40.4 | 2.66 | 1.02 | 2.65 | 0.59 | 2.91 | 1.01 | 3.03 | 0.86 | 0.0375 | 0.2856 |
| 14 | PC.aa.C32.0 | 11.90 | 3.16 | 11.55 | 2.51 | 12.46 | 3.21 | 12.45 | 2.81 | 0.0449 | 0.2958 |
| 15 | PC.ae.C42.3 | 0.46 | 0.13 | 0.51 | 0.12 | 0.49 | 0.13 | 0.55 | 0.10 | 0.0449 | 0.2958 |
| 16 | PC.ae.C40.1 | 0.76 | 0.35 | 0.85 | 0.23 | 0.83 | 0.31 | 1.01 | 0.19 | 0.0478 | 0.2958 |
| Supplemental Table 1F. Repeated measures ANOVA of the metabolite concentrations for significantly altered metabolites grouped by “Creatinine > 1.2mg/dl vs creatinine <1.2mg/dl” at time T0 and T1. Data are presented as mean concentration [µM] ± standard deviation. FDR, false discovery rate. Crea, Creatinine. P values are given for mean changes from T0 vs. T1 according to the outlined subgroup. | | | | | | | | | | | |

|  |  | **nt-proBNP** | | | | | | | |  |  |
| --- | --- | --- | --- | --- | --- | --- | --- | --- | --- | --- | --- |
|  |  | **T0** | | | | **T1** | | | |  |  |
|  |  | **BNP ≥ 1038ng/l (N=21)** | | **BNP < 1038ng/l (N=23)** | | **BNP ≥ 1038ng/l (N=21)** | | **BNP < 1038ng/l (N=23)** | | **rANOVA** | |
| **Rank** | **Metabolite** | **Mean conc. [µM]** | **SD** | **Mean conc. [µM]** | **SD** | **Mean conc. [µM]** | **SD** | **Mean conc. [µM]** | **SD** | **p-Value** | **FDR** |
| 1 | PC.aa.C36.3 | 72.98 | 20.17 | 78.98 | 26.04 | 82.26 | 24.29 | 87.73 | 18.41 | 0.0008 | 0.0792 |
| 2 | PC.ae.C36.4 | 11.63 | 2.84 | 12.62 | 4.51 | 12.69 | 3.50 | 13.33 | 3.04 | 0.0053 | 0.2624 |
| 3 | PC.aa.C28.1 | 2.21 | 0.74 | 2.35 | 0.56 | 2.53 | 0.87 | 2.70 | 0.67 | 0.0107 | 0.3174 |
| 4 | PC.aa.C32.1 | 14.25 | 6.28 | 15.94 | 10.07 | 16.29 | 8.29 | 19.34 | 14.49 | 0.0131 | 0.3174 |
| 5 | PC.aa.C32.0 | 11.67 | 2.62 | 11.66 | 2.85 | 12.50 | 3.14 | 12.40 | 2.75 | 0.0161 | 0.3174 |
| 6 | PC.aa.C40.4 | 2.62 | 0.64 | 2.68 | 0.84 | 2.99 | 0.89 | 2.99 | 0.93 | 0.0213 | 0.3174 |
| 7 | SM..OH..C22.1 | 7.28 | 2.56 | 8.18 | 1.71 | 7.81 | 2.67 | 8.92 | 2.00 | 0.0288 | 0.3174 |
| 8 | PC.ae.C38.3 | 2.88 | 0.76 | 3.14 | 0.68 | 3.04 | 0.70 | 3.34 | 0.72 | 0.0309 | 0.3174 |
| 9 | PC.ae.C40.1 | 0.77 | 0.30 | 0.87 | 0.25 | 0.89 | 0.27 | 1.01 | 0.21 | 0.0334 | 0.3174 |
| 10 | SM.C18.0 | 20.91 | 7.26 | 20.23 | 5.05 | 19.49 | 5.71 | 19.50 | 5.36 | 0.0355 | 0.3174 |
| 11 | PC.aa.C40.2 | 0.25 | 0.05 | 0.26 | 0.09 | 0.28 | 0.07 | 0.28 | 0.05 | 0.037 | 0.3174 |
| 12 | PC.ae.C32.1 | 2.11 | 0.51 | 2.07 | 0.62 | 2.21 | 0.59 | 2.20 | 0.69 | 0.0412 | 0.3174 |
| 13 | PC.aa.C36.6 | 0.44 | 0.20 | 0.57 | 0.23 | 0.49 | 0.20 | 0.66 | 0.23 | 0.0452 | 0.3174 |
| 14 | PC.aa.C42.6 | 0.31 | 0.08 | 0.31 | 0.08 | 0.32 | 0.05 | 0.33 | 0.08 | 0.0478 | 0.3174 |
| Supplemental Table 1G. Repeated measures ANOVA of the metabolite concentrations for significantly altered metabolites grouped by “BNP>1038ng/l vs. BNP<1038ng/l” at time T0 and T1. Data are presented as mean concentration [µM] ± standard deviation. FDR, false discovery rate. BNP, N-terminus pro-B type natriuretic peptide. P values are given for mean changes from T0 vs. T1 according to the outlined subgroup. | | | | | | | | | | | |
